# Urine-derived podocytes from steroid resistant nephrotic syndrome patients as a model for renal-progenitor derived extracellular vesicles effect and drug screening

**DOI:** 10.21203/rs.3.rs-3959549/v1

**Published:** 2024-02-28

**Authors:** Adele Tanzi, Lola Buono, Cristina Grange, Corinne Iampietro, Alessia Brossa, Fanny Oliveira Arcolino, Maddalena Arigoni, Raffaele Calogero, Laura Perin, Silvia Deaglio, Elena Levtchenko, Licia Peruzzi, Benedetta Bussolati

**Affiliations:** University of Turin: Universita degli Studi di Torino; University of Turin: Universita degli Studi di Torino; University of Turin: Universita degli Studi di Torino; University of Turin: Universita degli Studi di Torino; University of Turin: Universita degli Studi di Torino; Amsterdam UMC - Locatie AMC: Amsterdam UMC Locatie AMC; University of Turin: Universita degli Studi di Torino; University of Turin: Universita degli Studi di Torino; Children’s Hospital Los Angeles; University of Turin: Universita degli Studi di Torino; Amsterdam UMC - Locatie AMC: Amsterdam UMC Locatie AMC; Azienda Ospedaliero Universitaria Città della Salute e della Scienza di Torino: Azienda Ospedaliero Universitaria Citta della Salute e della Scienza di Torino; Universita degli Studi di Torino

**Keywords:** Urine-derived podocytes, steroid-resistant nephrotic syndrome, personalized therapy, extracellular vesicles, exosomes, permeability, Alport syndrome, renal progenitor cells, sumoylation, disease models

## Abstract

**Background:**

Personalized disease models are crucial for assessing the specific response of diseased cells to drugs, particularly novel biological therapeutics. Extracellular vesicles (EVs), nanosized vesicles released by cells for intercellular communication, have gained therapeutic interest due to their ability to reprogram target cells. We here utilized urinary podocytes obtained from children affected by steroid-resistant nephrotic syndrome with characterized genetic mutations as a model to test the therapeutic potential of EVs derived from kidney progenitor cells.

**Methods:**

EVs were isolated from kidney progenitor cells (nKPCs) derived from the urine of a preterm neonate. Three lines of urinary podocytes obtained from nephrotic patients’ urine and a line of Alport patient podocytes were characterized and used to assess albumin permeability in response to various drugs or to nKPC-EVs. RNA sequencing was conducted to identify commonly modulated pathways.

**Results:**

Podocytes appeared unresponsive to pharmacological treatments, except for a podocyte line demonstrating responsiveness, in alignment with the patient’s clinical response at 48 months. At variance, treatment with the nKPC-EVs was able to significantly reduce permeability in all the steroid-resistant patients-derived podocytes as well as in the line of Alport-derived podocytes. RNA sequencing of nKPC-EV-treated podocytes revealed the common upregulation of two genes (small ubiquitin-related modifier 1 (SUMO1) and Sentrin-specific protease 2 (SENP2)) involved in the SUMOylation pathway, a process recently demonstrated to play a role in slit diaphragm stabilization. Gene ontology analysis on podocyte expression profile highlighted cell-to-cell adhesion as the primary upregulated biological activity in treated podocytes.

**Conclusions:**

nKPCs emerge as a promising non-invasive source of EVs with potential therapeutic effects on podocyte dysfunction. Furthermore, our findings suggest the possibility of establishing a non-invasive in vitro model for screening regenerative compounds on patient-derived podocytes.

## Introduction

Podocytes are highly dynamic and terminally differentiated renal cells that act as the final barrier to proteins during glomerular blood filtration, thus playing a pivotal role in controlling glomerular permeability (1). Damage to podocytes can result in idiopathic nephrotic syndrome, a common pathology in children, with an annual incidence of 2–7 cases per 100,000 children. Genetic alterations in structural or secreted podocyte proteins characterize resistance to common treatments (steroid-resistant nephrotic syndromes) (2–4) and progression to end-stage kidney disease (5) among nephrotic syndrome patients.

During nephrotic syndrome, podocyte injury is marked by changes in morphology, such as vacuolization, loss of slit diaphragm proteins, and possible detachment from the glomerular basement membrane (6). Focal detachment of podocytes is observed in cases of massive proteinuria in both experimental (7) and human diseases (8). In this context, the isolation of podocytes from urine holds increasing interest for characterizing their pathologic features and identifying drug responses or other therapeutic strategies (9). Along this line, we recently isolated and characterized podocytes from patients with Alport syndrome (10),

Extracellular vesicles (EVs), cell-released vesicles involved in cell-to-cell communication, are gaining attention as biological tools with regenerative potential (11). In renal pathology, EVs derived from stem cells of different sources exert anti-apoptotic, anti-inflammatory, and pro-angiogenic effects, possibly through the transfer of mRNAs, miRNAs, and proteins to renal cells (12–15). We recent demonstrated the beneficial effect of mesenchymal stromal cell-derived EVs (MSC-EVs) on podocyte injury in an *in vitro* millifluidic model of the glomerular filtration barrier (16). Similarly, EVs derived from endothelial progenitor cells inhibited complement-induced podocyte apoptosis, nephrin shedding, and maintained permselectivity during inflammatory damage (17). However, the potential effect of stem cell-derived EVs on podocytes affected by genetic alterations remains unexplored. In this study, we hypothesized that EVs derived from neonatal kidney progenitor cells (nKPCs) could exhibit specific beneficial potential on podocytes, given their renal origin. These cells, isolated from preterm neonatal urine were previously reported to express embryonic kidney transcription factors, including SIX2 and CITED1 (18), and were shown to protect renal cells from hypoxic damage in a model of kidney graft perfusion (19).

In this study, we used podocytes with genetic alterations obtained from the urine of children suffering from steroid-resistant nephrotic syndrome to assess the effect and possible mechanisms of nKPC-EVs in comparison to common drugs known to modulate podocyte permeability. Urinary conditionally immortalized podocytes from an Alport patient were also used as an additional model of genetically diseased podocytes with altered permeability.

## Methods

### Ethical statement

All the enrolled subjects provided informed written consent. The study protocol was approved by the Bioethics Committee of the A.O.U. Città della Salute e della Scienza Hospital (protocol no. 0021671). The study was conducted according to the principles expressed by the Declaration of Helsinki of 1975, as revised in 2013.

### Patients

A total of three patients diagnosed with steroid-resistant nephrotic syndrome were recruited in this study. Genetic and clinical features of patients are described in Table 1. During follow up patient 1 and 2 did not respond to all the treatments attempted, progressed to CKD5 and were successfully transplanted without recurrence. Patient 3, which initially proved to be steroid resistant, had a late satisfactory response to steroids combined with angiotensin-converting enzyme inhibitors and one single dose of Rituximab, and after 48 months of follow up has a normal kidney function and minimal proteinuria.

### Generation and characterization of podocyte cell lines

The urines of the three patients with steroid-resistant nephrotic syndrome and of a healthy pregnant woman (Table 1) were freshly collected and centrifuged at 200 × g for 10 min. The pellet was resuspended in DMEM/F-12 (Life Technologies, Carlsbad, CA, USA) supplemented with 10% foetal calf serum (FCS; Invitrogen, Carlsbad, CA, USA), 50 IU/ml penicillin, 50 g/ml streptomycin, 5 mM glutamine, 5 g/ml insulin, 5 g/ml transferrin, and 5 mg/ml selenium (all from Sigma-Aldrich, St Louis, MO, USA). Subsequently, primary cells were grown at 37 °C up to the third passage and characterized as podocytes (NS-POD1, NS-POD2, NS-POD3 and CTL-uPOD, respectively). In addition, a conditionally immortalized podocyte cell line (AS-POD) from an Alport syndrome patient was used (10). Finally, tissue-derived podocytes, kindly gifted by MA Saleem, were used as an additional control for selected experiments (CTL-tPOD) (20).

### Generation and characterization of nKPC cell line

Urine sample (500 mL) was collected from a newborn (born at 32 gestational weeks) at day 1 after birth from a catheter bag, as described (18). The sample was centrifugated at 1’500 rpm for 10 min and the cell pellet was resuspended in α MEM Medium (Gibco/BRR ThermoFisher, MA, USA) supplemented with 20% Chang Medium B (Irvine Scientific, Santa Ana, California, USA) and 2% Chang Medium C (Irvine Scientific), 20% FCS (Invitrogen, Carlsbad, CA, USA), 50 IU/mL penicillin, 50 g/mL streptomycin, 5 mM glutamine (all from Sigma-Aldrich, St. Louis, MO, USA). At passage 2, 1 × 10^4^ primary nKPCs were infected in DMEM F12 20% FCS with a retrovirus containing a pBABE-puro-hTERT plasmid (Addgene plasmid #1771) (21). The day after the infection, the medium was replaced with their growth medium. From passage 3, cells were selected in their growth medium containing 1 μg/mL puromycin (Gibco, Thermo Fisher Scientific, Waltham, MA, USA) for four weeks, until passage 7.

### Isolation of nKPC-EVs

EVs were obtained from nKPC supernatant. The cells were cultured overnight in RPMI deprived of FCS, then the supernatant was centrifuged for the removal of cell debris and apoptotic bodies at 3,000 g for 20 min. EVs were purified by a 2 h-ultracentrifugation at 100,000 g at 4 °C (Beckman Coulter, Brea, CA, USA) and used fresh or stored at −80 °C after resuspension in RPMI supplemented with 1% dimethyl sulfoxide. Analysis of the size distribution and particle quantification were performed using NanoSight NS300 (NanoSight Ltd, Malvern, UK) equipped with a 405 nm laser and the Nanoparticle Tracking Analysis (NTA) 2.3 software (NanoSight Ltd., Malvern, UK). MSC and serum-derived EVs were isolated as previously described (16, 22).

### Protein extraction and Western Blot

For protein analysis, podocytes, nKPCs and nKPC-EVs were lysed at 4 °C for 20 min in RIPA buffer (20 nM Tris·HCl, 150 nM NaCl, 1% deoxycholate, 0.1% SDS 1% Triton X-100, pH 7.8) supplemented with protease and phosphatase inhibitor cocktail and PMSF (Sigma-Aldrich, St. Louis, MO, USA). To determine total protein concentration of podocyte lysate, Bradford solution was used, according to the manufacturer’s procedures (Bio-Rad Inc, Berkeley, CA, USA). At variance, total protein concentration of nKPCs and nKPC-EVs was determined spectrophotometrically using a micro-BCA^™^ Protein Assay Kit, as previously described (23). Either 30 μg (for podocytes) or 8 μg (for nKPCs and nKPC-EVs) of proteins were electrophoresed through 4–12% Mini-Protean TGX Stain-Free Gels (Bio-Rad). Using the iBLOT2 system (Life Technologies, Carlsbad, CA, USA), gels were blotted onto PVDF membrane filters according to the manufacturer’s procedures. Each membrane was immersed in blocking solution, consisting in 5% bovine serum albumin (BSA; Sigma-Aldrich, St. Louis, MO, USA) in PBS, for 1 h before overnight incubation with primary antibodies at the indicated dilutions. After rinsing in wash buffer (0.1% Tween in PBS), horseradish peroxidase-conjugated secondary antibodies were used for 1 h at 1:3000–1:5000 dilutions. After final washings, membranes were incubated with ECL chemiluminescence reagent (Bio-Rad). Images were acquired using a ChemiDoc ^™^ XRS+ System (BioRad, Milan, Italy). For podocytes analysis, the following antibodies were used: rabbit monoclonal anti-Podocin (Cat. No. sc-21009; Santa-Cruz, Dallas, TX, USA), and mouse monoclonal anti-CD2AP (Cat. No sc-25272, Santa-Cruz). Goat monoclonal anti-Vinculin (Cat. No. sc-7648 Santa-Cruz) was used as housekeeping. In the case of nKPCs and nKPC-EVs protein analysis, mouse monoclonal anti-CD63 (Cat. No sc-5275, Santa-Cruz) and rabbit monoclonal anti-Calreticulin (Cat. No 2891, Cell signalling, Milan, Italy) were used. The protein bands were detected using either rabbit, mouse, or goat peroxidase-labeled secondary antibody.

### Immunofluorescence

Immunofluorescence on podocytes was performed as follows: cells were fixed in 4% paraformaldehyde for 20 min at room temperature and permeabilized with PBS 0.1% Triton X-100 (Sigma-Aldrich, St. Louis, MO, USA) for 10 min at 4 °C. PBS 1.5% BSA (Sigma-Aldrich, St. Louis, MO, USA) was used to block non-specific sites for 20 min at room temperature. Subsequently, Texas Red-X Phalloidin (Cat. No. T7471; Thermo Fisher Scientific) was incubated for 1 h. Fixed cells were washed with PBS 0.1% BSA before nuclear staining with 4.6-diamidine-2-phenylindole (DAPI, Sigma-Aldrich) for 8 min. After the final wash, coverslips were mounted with Fluoromount. Images were acquired by the videoconfocal system ViCo microscope Nikon Eclipse 80i (Nikon, Japan).

### Transmission Electron Microscopy

The transmission electron microscopy (TEM) was performed on EVs placed on 200-mesh nickel formvar carbon-coated grids (Electron Microscopy Science) for 20 min to promote adhesion. The grids were then incubated with 2.5% glutaraldehyde plus 2% sucrose. EVs were negatively stained with NanoVan (Nanoprobes, Yaphank, NY, USA) and observed using a Jeol JEM 1400 Flash electron microscope (Jeol, Tokyo, Japan) (24).

### Super resolution microscopy

Super-resolution microscopy was performed with Nanoimager S Mark II microscope from ONI (Oxford Nano- imaging, Oxford, UK) equipped with a 100x, 1.4NA oil immersion objective, an XYZ closed-loop piezo 736 stage, and triple emission channels split at 640, 555 and 488 nm on nKPC-EV. EV profiler Kit (EV-MAN- 1.0, ONI) was used for the experiments following manufacturer’s protocol. The Kit contains fluorescent antibodies, anti CD9–488, CD63–568 and CD81–647, washing buffer and the imaging buffer. Images were acquired sequentially in dSTORM mode in total reflection fluorescence (TIRF). Single-molecule data was filtered using NimOS software (v.1.18.3, ONI). Data analysis was conducted using Collaborative Discovery (CODI) online analysis platform www.alto.codi.bio from ONI and the drift correction pipeline version 0.2.3 was used (25).

### RNA Isolation and Real Time PCR

Total RNA of podocytes untreated or treated for 24 h with nKPC-EVs (5×10^4^ EV/cell) was isolated using Trizol Reagent (Ambion, Austin, TX, USA) according to the manufacturer’s protocol. At variance, RNA of nKPCs and of nKPC-EVs was extracted using miRNeasy mini kit (Qiagen GmbH, Qiagen Strasse 1, 40724 Hilden, Germany) according to the manufacturer’s protocol. RNA was then quantified spectrophotometrically (Nanodrop ND-1’000, Wilmington, NC, USA). For the gene expression analysis, quantitative real-time PCR (RT-PCR) was performed. Briefly, one-strand cDNA was produced from 200 ng of total RNA using a High-Capacity cDNA Reverse Transcription Kit (Applied Biosystems, Waltham, MA, USA). RT-PCR experiments were performed in a 20 μL-reaction mixture containing 5 ng of cDNA template, the sequence-specific oligonucleotide primers (purchased from MWG-Biotech, Eurofins Scientific, Brussels, Belgium), and the Power SYBR Green PCR Master Mix (Applied Biosystems, Waltham, MA, USA). GAPDH mRNA was used to normalize the RNA inputs. The fold change expression with respect to the control was calculated for all the samples. Primer list can be found in supplementary data (Supplementary table I).

### Flow cytometry

After puromycin selection, nKPCs were detached using a nonenzymatic cell dissociation solution, resuspended in PBS 0.1% BSA (Sigma-Aldrich, St. Louis, MO, USA) and incubated with antibodies. Cells were incubated with either phycoerythrin (PE)-, fluorescein isothiocyanate (FITC)-, or allophycocyanin (APC)-conjugated antibodies against CD90 (Cat. No. 130-114-859, Miltenyi Biotec, Bergisch Gladbach, Germany) CD73 (Cat. No. 550257, BD Bioscience, Franklin Lakes, NJ, USA), CD146 (Cat. No. 550315, BD Bioscience) and CD29 (Cat. No. 130-101-256, Miltenyi Biotec) CD133-1 (Cat. No. 130-090-826, Miltenyi Biotec) and appropriate isotype control. Stained cells were then analyzed using FACSCalibur machine using CellQuest software (Becton Dickinson Bioscience Pharmingen, Franklyn Lake, NJ, USA).

### MACSPlex Analysis

nKPC-EVs were subjected to bead-based multiplex EV analysis by flow cytometry (MACSPlex Exosome Kit, human, Miltenyi Biotec, Auburn, CA, USA), as previously described (25). Briefly, 5´10^9^ EVs were diluted with a MACSPlex buffer (MPB) to a final volume of 120 μL and 15 μL of MACSPlex Exosome Capture Beads (containing 39 different antibody-coated bead subsets) were added to each sample. The samples were then incubated on an orbital shaker overnight (14–16 h) at 450 rpm at 4 °C protected from light, followed by several washings with MPB using centrifugations (3’000 g, 5 min). For EV counterstaining, 5 mL of each APC-conjugated anti-CD9, anti-CD63, and anti-CD81 detection antibodies were added to each sample and incubated on an orbital shaker at 450 rpm for 1 h at room temperature. After additional washings, samples were subjected to flow cytometric analysis using FACS Celesta (BD Biosciences, Franklin Lakes, NJ, USA).

### Permeability assay

Permeability assays were performed by measuring BSA filtration from the lower to the upper compartment, after 24-hour treatments. Complete medium (500 mL) containing or not FITC-BSA (1 mg/mL, Sigma Aldrich) was placed in the lower compartment and upper podocyte compartments, respectively. To measure the podocyte filtration ability in basal to apical direction, 100 μl of medium was taken after 6 h from the upper compartment and the passage of FITC-BSA was determined by fluorimetry in triplicate. FITC signal was measured in triplicates using Promega^™^ GloMax^®^ Plate Reader (Promega Italia S.r.l., Milano, Italy). Data are expressed as the mean amount of filtered BSA-FITC of four different experiments using at least three inserts for each condition in each experiment. Podocytes were treated for 24 h with nKPC-EVs (2–5 ×10^4^ EV/cell), methylprednisolone (Urbason, Sanofi 40 mg/mL), cyclosporin (Sandimmun, Novartis 25 mg/mL), tacrolimus (Prograf, Panacea Biotec, 1 ng/mL) and rituximab (Mabthera, Roche, 12 mg/mL).

### RNA sequencing analysis

Total RNA from untreated or treated podocytes was isolated using Trizol Reagent (Ambion, Austin, TX, USA) according to the manufacturer’s protocol. RNA was then quantified spectrophotometrically (Nanodrop ND-1’000, Wilmington, NC, USA). Libraries for RNA-seq were generated using a TruSeq RNA stranded sample preparation kit v2 (Illumina Inc, San Diego, CA, USA) following the manufacturer’s instructions, using 1 μg of total RNA as input material. Libraries were pooled and sequenced with a NextSeq 500 sequencer (Illumina Inc) generating 75-bp paired-end sequences. Further analyses were performed using transcript per million (TPM) tables and genes with an average |log_2_ Fold Change| 31 were considered for further analysis using Expression Suite and Funrich V3 Software (Bundora, Australia).

### Statistical analysis

Data are shown as mean ± SD. Statistical analysis was carried out on Graph Pad Prism (GraphPad Software, Inc., San Diego, CA, USA) by using one-way analysis of variance (ANOVA) followed by Dunnet’s multiple comparisons test, or by unpaired t-test, where appropriate. A p value < 0.05 was considered significant.

## Results

### Podocyte isolation and characterization

Podocytes were obtained from freshly collected urine derived from three different patients presenting steroid-resistant nephrotic syndrome (NS-POD). Genetically characterized mutations are described in Table I. Podocytes isolated from healthy pregnant woman’s urine were used as control (CTL-uPOD).

**Figure 1:**
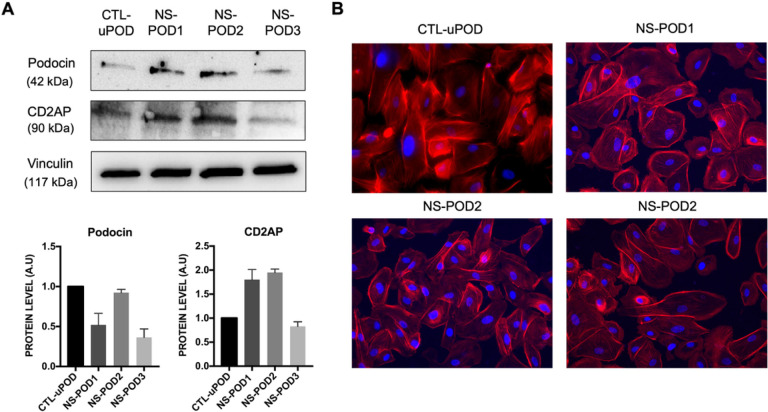
Isolation and characterization of podocytes from urine. **A.** Western blot analysis (representative images and quanti cation) of podocytes derived from urine of three different patients (NS-POD1, NS-POD2, NS-POD3) and of a healthy pregnant woman (CTL-uPOD) positive for podocin and CD2AP. Data are expressed as mean of two experiments ± SD. **B**. Representative micrographs of podocytes deriving from urine of CTL-uPOD, NS-POD1, NS-POD2 and NS-POD3 stained with phalloidin (red) and blue nuclear stain DAPI. Original magnfi cation: X20.

Urine-derived cells were characterized by the presence of podocyte markers such as podocin and CD2AP (Fig. 1A) and by morphology, showing cells with an organized cytoskeletal structure similar to already well-characterized podocytes (Fig. 1B) (10).

The podocyte phenotype was also assessed by analyzing the expression of a specific signature composed of 68 podocyte-typical genes (Supplementary Fig. 1), as previously outlined by Lu et al., in comparison to normal tissue podocytes (CTL-tPOD) (10, 26). All three podocyte lines expressed the podocyte signature, with no significant differences in their expression profile compared to that of tissue podocytes, thereby confirming the podocyte phenotype (Supplementary Fig. 1) (10). Additionally, conditionally immortalized podocytes, isolated from the urine of a patient with Alport syndrome and previously characterized in our laboratory (10), were included in the study (AS-POD) (Table I).

### Kidney progenitor-derived EVs isolation and characterization

**Figure 2:**
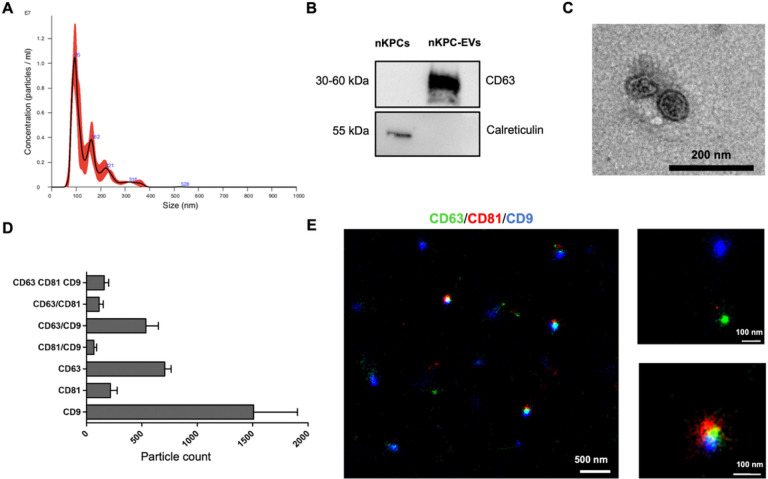
Characterization of renal progenitor-derived EVs. **A**. Representative NTA analysis showing the EVs size distribution; EVs mean size is 142.7 nm. **B.** Representative western blot image showing the presence of CD63 in nKPC-EVs (8 μg proteins) and the presence of calreticulin in cell lysate only. **C.** Representative micrograph of TEM of nKPC-EVs (scale bar: 200 nm). **D.** Clustering analysis of super-resolution microscopy images shows the single, double, and triple positive EV fractions expressing the tetraspanins. The analyses were performed in 3 nKPC-EV preparations using a CODI software; the graph shows the mean ± SD of a cumulative analysis of 3 fields for each preparation. **E.**Representative super-resolution microscopy images of nKPC-EVs showing expression of CD63 (green), CD81 (red) and CD9 (blue). The scale bares are below each EV image.

EVs were isolated by differential centrifugations from immortalized nKPCs. These cells were originally obtained from the urine of a preterm infant and characterized as previously described (18). The size distribution of nKPC-derived EVs was analyzed using nanosight tracking analysis (NTA), revealing a mean size distribution of approximately 142.7 nm (Fig. 2A). Western Blot analysis con rmed the presence of the EV-specific marker CD63, while the absence of the cytoplasmic marker calreticulin indicated the absence of cellular contamination (Fig. 2B). Evaluation by TEM revealed the typical cup-shaped morphology of these EVs (Fig. 2C). Additionally, super-resolution microscopy demonstrated that nKPC-EVs, stained for CD9, CD63, and CD81 expression, expressed tetraspanins, typical EV markers, in various combinations, predominantly being CD63 and CD9 positive (Fig. 2D–E).

**Figure 3:**
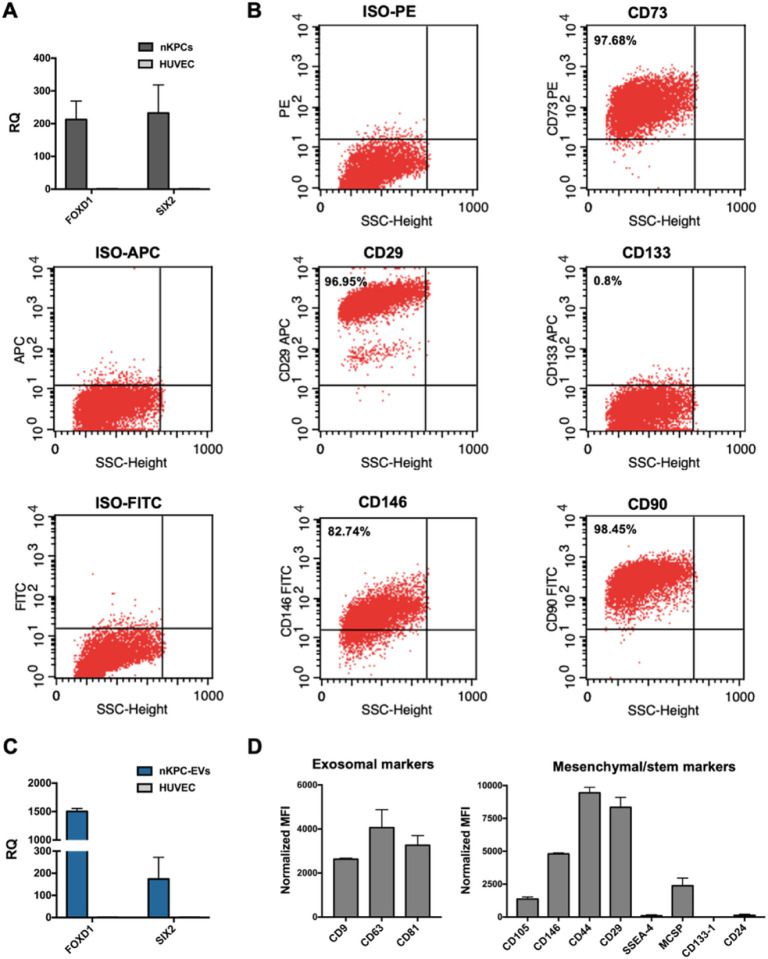
nKPC and nKPC-derived EV characterization of intracellular and surface markers expression. **A.** RT-PCR analysis showing the expression of FOXD1 and SIX2 in nKPCs. GAPDH was used as an endogenous normalizer. Data were further normalized to HUVEC cells, used as a negative control for each experiment. The graphs show the RQ average (2^−ΔΔCt^) of three independent experiments ± SD. B. Representative flow cytometry dot plot of nKPCs showing positive expression of CD73, CD90, CD29, CD146 and resulted to be negative for CD133. **C.** RT-PCR analysis showing the expression of FOXD1 and SIX2 in nKPC-EVs. GAPDH was used as an endogenous normalizer. Data were further normalized to HUVEC cells, used as a negative control for each experiment. The graphs show the RQ average (2^−ΔΔCt^) of two independent experiments ± SD. **D.** Quantification of the median APC fluorescence for each bead population after background correction of exosomal and mesenchymal/stem cell markers in nKPC-EVs. The fluorescence intensity of each marker was normalized to the mean fluorescence intensity of all detectable markers to 1000. Data are expressed as the average of two technical replicates ± SD.

The expression of mesenchymal and renal progenitor markers in nKPCs and deriving EVs was also evaluated. Notably, nKPCs exhibited a mesenchymal phenotype, expressing CD90, CD73, CD146, and CD29 (Fig. 3B), along with the nephron progenitor marker SIX2 and stromal progenitor marker FOXD1 (Fig. 3A), as previously reported (18). Conversely, nKPCs were negative for markers associated with adult renal progenitor cells, such as CD133 (Fig. 3B) (27). Similarly, using a bead-based immunocapture assay, nKPC-EVs were found to express mesenchymal stromal markers CD146, CD29, and CD44, in addition to the typical EV markers (CD9, CD63, and CD81), while showing no expression of CD133 or stage-specific embryonic antigen-4 (SSEA-4; Fig. 3D). Furthermore, nKPC-EVs demonstrated the presence of SIX2 and FOXD1 mRNAs, as confirmed by RT-PCR (Fig. 3C).

### Permeability analysis of podocyte cultures

**Figure 4:**
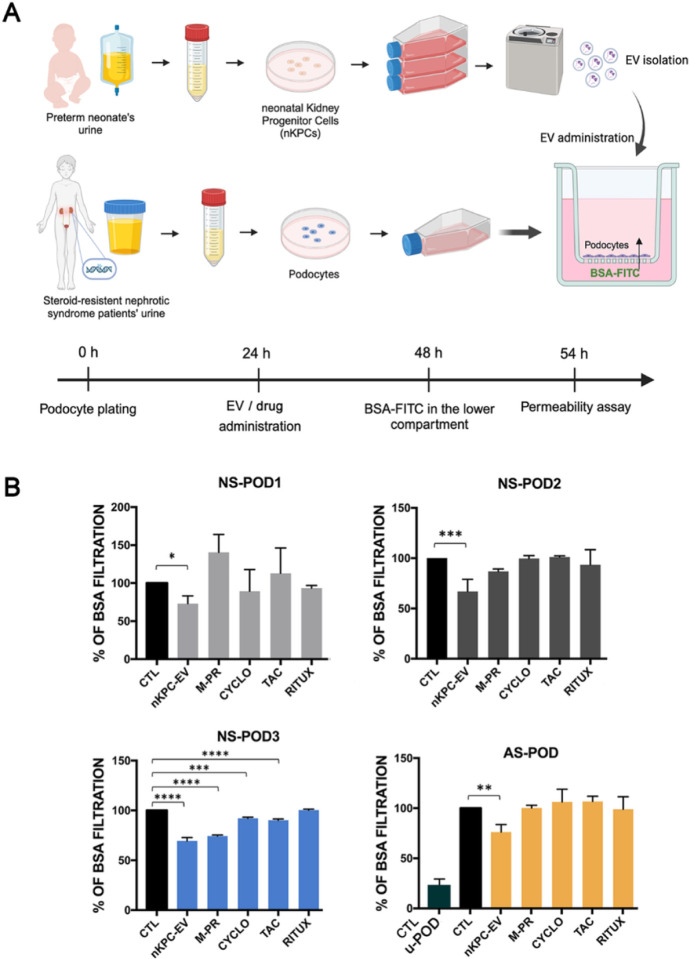
Permeability assay in podocyte cultures treated with nKPC-EVs. A. Schematic experimental design and graphic representation of culture set-up. B. Graphical representation of FITC-BSA permeability indicating albumin passage from the lower compartment to the upper compartment for urinary podocyte lines (NS-POD: nephrotic syndrome podocytes, AS-POD: Alport syndrome podocytes, CTR-uPOD: control urinary podocytes). Untreated cell condition was used as control for each experiment, set as BSA filtration rate of 100%. and compared to FITC-BSA filtration percentages of podocytes treated with nKPC-EVs (5×10^4^ EVs/cell) and with the different drugs methylprednisolone (M-PR, 40 mg/mL), cyclosporin (CYCLO, 25 mg/mL), tacrolimus (TAC 1 ng/mL) and rituximab (RITUX 12 mg/mL). Data are expressed as the mean amount of filtered BSA-FITC of four different experiments using at least three inserts for each condition in each experiment. One-way ANOVA with Dunnett’s multiple comparisons test was performed after the normalization of each experiment to untreated podocytes; * p < 0.05, ** p < 0.01, *** p < 0.001, **** p < 0.0001, vs the respective untreated condition.

To evaluate whether nKPC-EVs could modulate permselectivity of the patient-derived podocytes, we used an *in vitro* permeability assay. Podocytes were seeded on the upper side of inserts and permeability was assessed by measuring the transit of FITC-BSA from the lower compartment to the upper podocyte compartment (Fig. 4A). nKPC-EV efficacy was compared to that of commonly used drugs for treating renal diseases: methylprednisolone (M-PR), cyclosporin (CYCLO), tacrolimus (TAC), and rituximab (RITUX). While these drugs primarily function through immunomodulation, there have been reports of a direct anti-proteinuric effect targeting podocytes (28–30).

Podocytes treatment for 24 hours with nKPC-EVs resulted in reduced permeability across all podocyte lines of nephrotic syndrome patients as well of Alport podocytes (Fig. 4B). No modulation of permeability by pharmacological treatment was observed in the genetically altered podocytes, except for NS-POD3 (Fig. 4B). Interestingly, after 48 months, the patient generating NS-POD3 cells clinically responded to treatment with steroids, cyclosporine and Rituximab (see methods and Table I), supporting the use of patient-derived podocytes as model for drug response. Permeability of normal podocytes (uPOD) was significantly lower in respect to diseased podocytes (Fig. 4B), as described (10).

**Figure 5:**
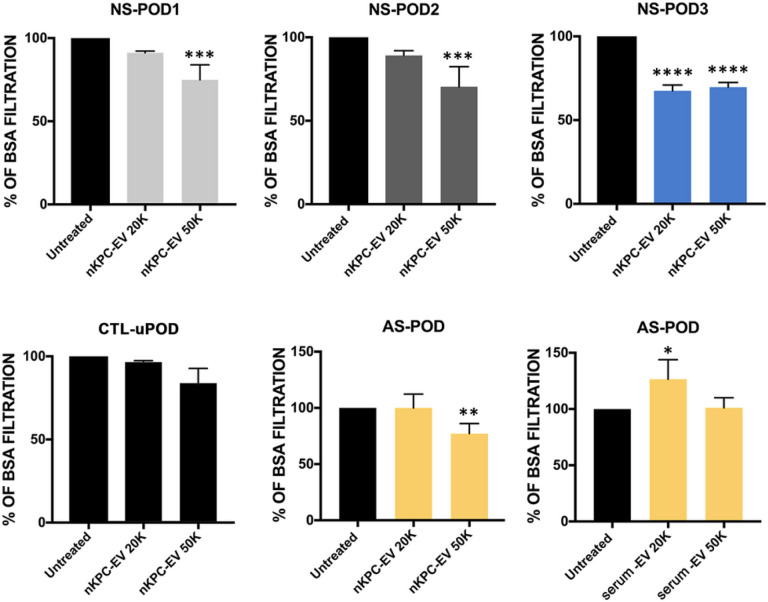
Permeability assay in podocyte cultures treated with nKPC-EVs and drugs. Untreated condition was compared to treatment with different doses of nKPC-EVs or serum-derived EVs (20K: 2 × 10^4^ EVs/cell, 50K: 5 × 10^4^ EVs/cell). NS-POD: nephrotic syndrome podocytes, AS-POD: Alport syndrome podocytes, CTR-uPOD: control urinary podocytes. Data are expressed as the mean amount of filtered BSA-FITC of four different experiments using at least three inserts for each condition in each experiment. One-way ANOVA with Dunnett’s multiple comparisons test was performed after the normalization of each experiment to untreated podocytes; * p < 0.05, ** p < 0.01, *** p < 0.001, **** p < 0.0001, vs the respective untreated condition.

The evaluate the dose response of podocytes to nKPC-EVs, experiments were performed using two doses (2 × 10^4^ and 5 × 10^4^ EVs/cell). nKPC-EVs were able to reduce permeability in all the different patient-derived podocytes, with 5×10^4^ EVs/cell dose resulting more effective (Fig. 5). At variance, no effect of serum-derived EVs was observed when tested on Alport podocytes. No effect was observed on control normal podocytes (CTL-uPOD) (Fig. 5).

### Transcriptomic analysis of urine-derived podocytes treated with EVs

**Figure 6:**
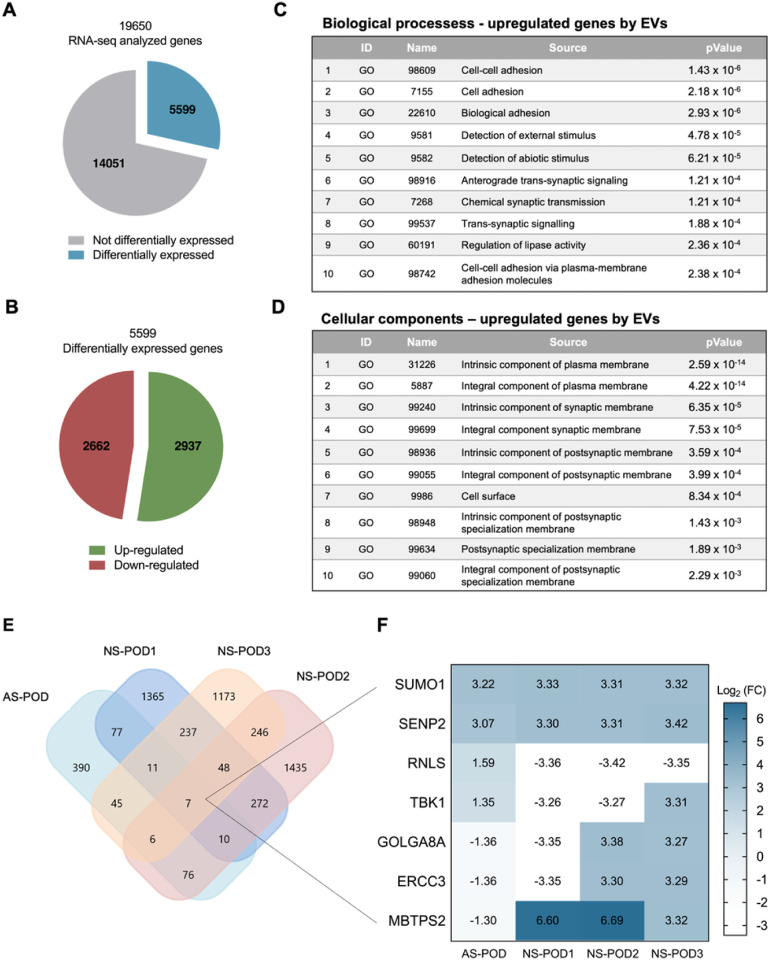
Cross-analysis for the identification of regulated genes common to the four patient-derived podocytes. **A.** Pie chart representing the sum of all the genes differentially expressed in the four different podocyte lines after the treatment with nKPC-EVs (in blue), compared with the non-differentially expressed genes (grey). **B.** Pie chart representation of up-regulated (green) and down-regulated (red) differentially expressed transcripts in the four different podocyte lines untreated and treated with EVs. **C and D.** Gene Ontology (GO) analysis of differentially expressed genes in the four different podocyte lines untreated and treated with EVs. In each table, the identification (ID) number, the name, and the P value associated with the GO are given. **E.** Representative Venn diagram showing the numbers of the genes that resulted up-regulated or down-regulated after the treatment of the podocytes with nKPC-EVs by total RNA sequencing analysis. Data were analyzed using Expression Suite and Funrich V3 Software. **F.** Heatmap showing the levels of expression regulation of the seven genes which were EV-regulated in all the four podocyte lines.

To explore the modifications in the transcriptome, we conducted total RNA sequencing on both untreated and nKPC-EV-treated podocytes. The transcripts per kilobase million (TPM) normalization method was employed to compare changes in the gene expression profile between the samples. Our analysis revealed a total of 2876 upregulated genes (log_2_ fold change >1) and 2574 downregulated genes (log_2_ fold change <−1) in response to EV treatment (refer to Fig. 6). Gene Ontology analysis highlighted cell-to-cell adhesion as the primary upregulated biological activity in treated podocytes. Moreover, seven genes exhibited regulation in all four samples post nKPC-EV treatment (refer to Fig. 6F), with only two genes, SUMO1 and SENP2, consistently regulated, showing a log_2_ fold change ranging between 3.07 and 3.42. Interaction network analysis demonstrated a direct interaction between these two upregulated genes (31, 32).

**Figure 7:**
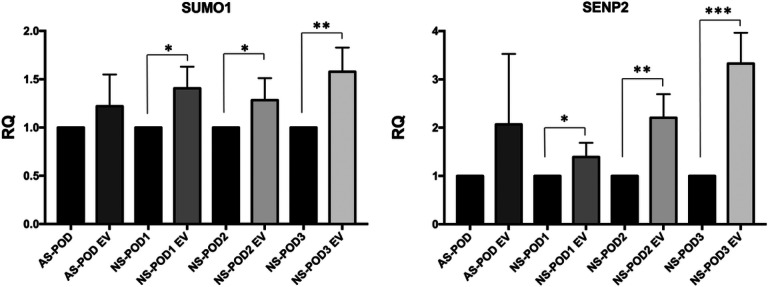
Validation of differentially expressed genes in urine-derived podocytes treated with EVs. mRNA expression of SUMO1 and SENP2 genes in urine-derived podocytes treated or not with nKPC-EVs. Data are shown as relative quantification, normalized to GAPDH and to each untreated control respectively set as 1. The graphs show the RQ average (2^−ΔΔCt^) of at least three independent experiments ± SD. Unpaired t-test was performed after the normalization of each experiment to its untreated condition (AS-POD, NS-POD1, NS-POD 2, NS-POD3, respectively); * p < 0.05, ** p < 0.001, *** p < 0.0001 vs AS-POD, NS-POD1, NS-POD 2, NS-POD3, respectively.

The nKPC-EV-induced up-regulation of both SUMO1 and SENP2 in urine-derived podocytes was confirmed by RT-PCR, where the up-regulation reached significance in three podocytes lines from nephrotic syndrome patients (Fig. 7). No effect on SUMO or SENP2 modulation was observed using MSC-EVs (not shown).

## Discussion

The current study provides a novel approach to directly assess the potential of regenerative therapies on patient-derived podocytes with genetic alterations. We successfully demonstrated the efficacy of extracellular vesicles (EVs) derived from neonatal renal progenitors in improving permeability control in podocytes obtained from patients with steroid-resistant nephrotic syndrome. Furthermore, we identified a potential mechanism involving the modulation of the SUMO pathway by nKPC-EVs by comparison of the data obtained in the patient specific lines.

The availability of human podocytes that accurately replicate human pathologies in culture is currently limited. Recently, podocyte-like cells have been successfully derived from induced pluripotent stem cells of patients, offering a promising avenue (33). However, obtaining a purely homogeneous population of podocytes presents challenges, as differentiated induced pluripotent stem cell cultures typically consist of only 30–50% of cells displaying podocyte-like morphology (34).

Another potential source for modeling diseases is the differentiation of urinary renal progenitor cells into podocytes, although this process may involve cell culture and differentiation stimuli (35). Human urine has been previously utilized as a non-invasive and valuable source of podocytes (8). In patients with active glomerular diseases, podocytes are shed from the glomerulus in response to local environmental factors. Patient-derived podocytes have been demonstrated to be positive for podocyte markers, viable, and capable of growth in culture (8). Conversely, podocytes derived from healthy subjects have been reported to exhibit limited replication potential in culture in the majority of cases (8). Of interest, increased podocyturia is observed in the urine of uncomplicated pregnant women (36), making it a valuable source for obtaining healthy control podocytes.

In this context, our in vitro model of glomerular permeability utilizing podocytes directly obtained from patients’ urine holds significant promise for personalized medicine. This model proved effective in evaluating the impact of drugs commonly administered to patients with steroid-resistant nephrotic syndrome. Notably, the drug response efficacy was observed specifically in the podocyte line from a patient who, despite being clinically steroid-resistant, exhibited subsequent clinical improvement with minimal proteinuria after 48 months. This personalized approach has the potential to set the stage for tests capable of predicting patient responses, guiding clinicians in treatment decisions, and avoiding unnecessary immunosuppressive interventions in the management of nephrotic syndrome. It must be however underlined the short survival of those primary lines, that only allow a restricted number of tests. This limitation prompted us to introduce in the study the conditionally immortalized Alport podocytes for additional control experiments.

Another potential application of the primary model of the glomerular filtration barrier was to evaluate the impact of a novel therapeutic approach centered on extracellular vesicles (EVs) derived from renal progenitor cells, directly on genetically mutated patient-derived podocytes. Our findings indicate that nKPCs could serve as a valuable source of EVs with regenerative potential. Interestingly, no effect on primary normal podocytes was observed, further supporting the importance of disease models for assessing therapeutic approaches

EVs, in particular those obtained from MSCs, have been previously studied in *in vitro* and *in vivo* experimental models of kidney disease (16, 37–41). Using a millifluidic model of glomerular filtration barrier, we previously demonstrated that MSC-EVs could traverse the basal membrane, reach podocytes, transfer their RNA cargo, resulting in protection against doxorubicin-induced injury (16). Other studies have underscored the protective roles of MSC-EVs on mouse podocyte lines within a model of diabetic nephropathy (40,41). However, the identification of specific mechanisms involved in the effect of EVs on target cells appears quite challenging, in consideration to the multitude of EV cargoes and to the patient-derived cell heterogeneity.

In our approach, we searched for common nKPC-EV-regulated genes at the transcriptomic level. Of interest, SUMO1 and SENP2, its modulator, were identified as the only common genes concordantly regulated in all patient-derived podocyte lines. At variance, no SUMO or SENP2 modulation was detected using MSC-EV treatment. The activity of SUMO, or SUMOylation, consists in a post-translational modification that alters the function of target proteins and modulates cell processes such as protein stability, localization, and activity (42,43). In podocytes, SUMO1 is considered to be involved in slit diaphragm protein stabilization, thus playing a possible role in the control of glomerular permeability (42). Since SENP2 is a known regulator of SUMO1 (30), it may be indirectly involved in slit diaphragm stabilization as well. Concordantly, Gene Ontology analysis highlighted cell-to-cell adhesion as the primary upregulated biological activity in nKPC-EV treated podocytes. Therefore, the potential effect of EVs derived from kidney progenitors on genetically altered podocytes represents a promising strategy for addressing genetic pathologies currently lacking a therapeutic solution. While this strategy was tested on three podocyte lines in this study, further validation and expansion to a larger patient population are warranted.

## Conclusion

In conclusion, we presented a human *in vitro* functional model capable of evaluating responses to common drugs and demonstrating the positive impact of nKPC-EVs on albumin permeability, thanks to combined effort of clinical, genetic and basic science. Our findings indicate that the administration of nKPC-EVs could significantly improve podocyte function. Furthermore, through total RNA sequencing, we identified the regulation of a SUMOylation pathway by nKPC-EVs, a pathway crucial for stabilizing podocyte slit diaphragm proteins.

Our data may pave the road for establishing a standard non-invasive *in vitro* model for the screening of regenerative compounds directly on patient-derived podocytes.

## Figures and Tables

**Table 1.** Genetic and clinical features of patients.

Patient	Sex	Gene	Variant	Zygosity	ACMG classification	Coded protein	Clinical follow-up after 48 months
NS-POD1	M	*NPHS2*	c.479A>G	Compound Heterozygous	C4	Podocin	Progression to CKD stage 5 and transplant
			c.855_856delAA	-	C5	Podocin	
NS-POD2	M	*PLCE1*	c.1405T>A	Compound Heterozygous	C1	Phospholipase C epsilon 1	Progression to CKD stage 5 and transplant
			C.3281 G>C	-	C3		
NS-POD3	F	LAMB2	c.4874_4876delTCC	Heterozygous	C3	Laminin beta-2 subunit	Clinical remission with proteinuria/creatininuria: <0.3 mg/mg.
AS-POD	F	COL4A3	c.2777del	Heterozygous	C4	Collagen alpha 3	Progression to CKD stage 5 and transplant

Abbreviations: F: female; M: male; CKD: chronic kidney disease.

## Data Availability

RNA sequencing data are available in the supplementary data.

## Abbreviations

EVs: Extracellular vesicles
nKPCs: neonatal kidney progenitor cells
NS-POD: nephrotic syndrome podocytes
AS-POD: Alport syndrome podocytes
CTL-uPOD: control urine podocytes
CTL-tPOD: control tissue podocytes
FCS: foetal calf serum
BSA: bovine serum albumin
NTA: nanoparticle tracking analysis
TEM: transmission electron microscopy
RT-PCR: real-time PCR
PE: phycoerythrin
FITC: fluorescein isothiocyanate
APC: allophycocyanin
MPB: MACSPlex buffer
CKD: chronic kidney disease
SSEA-4: stage-specific embryonic antigen-4
M-PR: methylprednisolone
CYCLO: cyclosporin
TAC: tacrolimus
RITUX: rituximab
MSCs: mesenchymal stromal cells
SUMO1: small ubiquitin-related modifier 1
SENP2: Sentrin-specific protease 2
